# Prognostic value of the triglyceride-glucose Index in elderly patients with acute decompensated heart failure: a one-year mortality analysis

**DOI:** 10.3389/fcvm.2026.1747197

**Published:** 2026-03-27

**Authors:** Ahmet Yilmaz, Orhan Çiçek

**Affiliations:** 1 Department of Cardiology, Karamanoğlu Mehmetbey University, Karaman, Türkiye; 2 Department of Geriatrics and Clinical Gerontology, Konya Sehir Hastanesi, Konya, Türkiye

**Keywords:** acute decompensated heart failure, geriatric patients, insulin resistance, mortality, prognosis, triglyceride-glucose index

## Abstract

**Background:**

The triglyceride-glucose (TyG) index, a simple surrogate marker of insulin resistance, has been associated with adverse cardiovascular outcomes. However, data on its prognostic value in elderly patients with acute decompensated heart failure (ADHF) are limited. This study aimed to evaluate the predictive role of the TyG index for one-year all-cause mortality in geriatric ADHF patients.

**Methods:**

This retrospective single-center study included 149 patients aged ≥65 years who were hospitalized with ADHF between January 2023 and December 2024. The TyG index was calculated as ln [fasting triglyceride (mg/dL) × fasting glucose (mg/dL)/2]. Patients were stratified into quartiles based on TyG levels, and outcomes were compared across groups. Receiver operating characteristic (ROC) curves, Kaplan–Meier survival analysis, and multivariate Cox regression were applied to evaluate the prognostic performance of the TyG index.

**Results:**

Higher TyG quartiles were significantly associated with increased glucose, triglyceride, and inflammatory marker levels (*p* < 0.05). ROC analysis demonstrated moderate discriminative ability for one-year mortality (AUC = 0.72, 95% CI: 0.61–0.83, *p* = 0.001), with an optimal cut-off value of 8.70. Kaplan–Meier curves showed significantly reduced survival in the highest TyG quartile (45.9%) compared with the lowest (89.5%) (log-rank *p* < 0.001). Although TyG was not an independent predictor in multivariate analysis (HR 1.68; 95% CI: 0.70–4.00; *p* = 0.25), it was significant in the subgroup with left ventricular ejection fraction <40% (HR 1.91; 95% CI: 1.02–3.59; *p* = 0.04). Adding the TyG index to a conventional risk model including age, sex, left ventricular ejection fraction, hypertension, chronic obstructive pulmonary disease, cardiovascular disease, and chronic kidney disease significantly improved prognostic accuracy (NRI = 0.273, IDI = 0.011, both *p* < 0.01).

**Conclusion:**

Elevated TyG levels are associated with increased one-year mortality and reduced survival in elderly ADHF patients. The TyG index provides incremental prognostic value beyond conventional risk factors and in daily clinical practice may serve as a simple, low-cost tool for risk stratification in geriatric heart failure management.

## Introduction

Heart failure (HF) remains one of the leading causes of morbidity and mortality worldwide, particularly among elderly populations in whom physiological reserve is diminished and comorbid metabolic disorders are common (1). Despite advances in pharmacological and device-based therapies, outcomes in patients with acute decompensated heart failure (ADHF) remain suboptimal, with one-year mortality rates frequently exceeding 25%–30% in older cohorts. Identifying simple and reliable biomarkers that can aid in early risk stratification is therefore crucial for early risk stratification (2).

Insulin resistance (IR) has been recognized as a pivotal metabolic disturbance contributing to both the development and progression of HF. Beyond its classical role in glucose homeostasis, IR promotes endothelial dysfunction, oxidative stress, myocardial remodeling, and neurohormonal activation all of which may exacerbate cardiac dysfunction (3). Although the hyperinsulinemic–euglycemic clamp remains the gold standard for measuring IR, its complexity and cost preclude widespread clinical use. As a result, surrogate indices have gained attention, among which the triglyceride-glucose (TyG) index has emerged as a simple, reproducible, and cost-effective alternative derived from fasting triglyceride and glucose levels. Fasting triglyceride levels ≥150 mg/dL are generally considered metabolically abnormal and prognostically relevant, especially when interpreted together with fasting glucose as part of the TyG index (4).

The triglyceride-glucose (TyG) index has been shown in multiple studies to be strongly associated with insulin resistance and to outperform HOMA-IR in predicting metabolic and cardiovascular risk (4, 5). Elevated TyG values have been associated with increased incidence of atherosclerotic cardiovascular disease, left ventricular (LV) remodeling, and HF onset in community-based populations (6). In the Atherosclerosis Risk in Communities (ARIC) study, higher baseline TyG index independently predicted new-onset HF and LV dysfunction over two decades of follow-up, underscoring its potential pathophysiological relevance (7).

Subsequent clinical investigations have extended these findings to patients with established HF. Zhou et al. (8) demonstrated that higher TyG tertiles were significantly associated with all-cause and cardiovascular mortality in chronic HF, improving model discrimination beyond conventional risk factors. A meta-analysis by Khalaji et al. (3), encompassing over 770,000 participants, confirmed that each unit increase in TyG index corresponded to a 17% higher risk of HF incidence and adverse outcomes. Similarly, Dou et al. (2) observed that lower triglyceride-glucose index combined with body mass index (TyG-BMI) values were linked to increased one-year mortality in hospitalized HF patients, highlighting the complex bidirectional interplay between metabolic status and cardiac outcomes.

Of particular relevance to geriatric cardiology, insulin resistance and its metabolic sequelae often intersect with frailty, sarcopenia, and systemic inflammation, compounding vulnerability in elderly individuals (9). The TyG index and its related measures [e.g., TyG-BMI, triglyceride-glucose index combined with waist-to-height ratio (TyG-WHtR)] have recently been shown to correlate with frailty indices, suggesting shared metabolic pathways underlying both conditions. Despite these insights, evidence specifically addressing the prognostic significance of the TyG index in elderly patients hospitalized with acute decompensated heart failure (ADHF) remains scarce. Most prior studies have focused on chronic HF populations or mixed-age cohorts, leaving a critical knowledge gap in older adults—who constitute the majority of ADHF admissions and exhibit the highest post-discharge mortality.

Given its simplicity and clinical practicality, the TyG index may serve as a low-cost, accessible adjunct to conventional prognostic models used in heart failure. Therefore, this study aimed to evaluate the prognostic value of the triglyceride-glucose index in elderly patients hospitalized with acute decompensated heart failure (ADHF), focusing on one-year all-cause mortality. By integrating both metabolic and cardiovascular risk dimensions, we hypothesized that a higher TyG index would be independently associated with increased one-year mortality among elderly ADHF patients.

## Methods

### Study design and population

This retrospective, single-center, observational cohort study was conducted at the Department of Cardiology, Karamanoğlu Mehmetbey University Faculty of Medicine, Karaman Training and Research Hospital (Karaman, Türkiye). The study aimed to investigate the prognostic significance of the TyG index in elderly patients hospitalized with acute decompensated heart failure.

**Figure 1:**
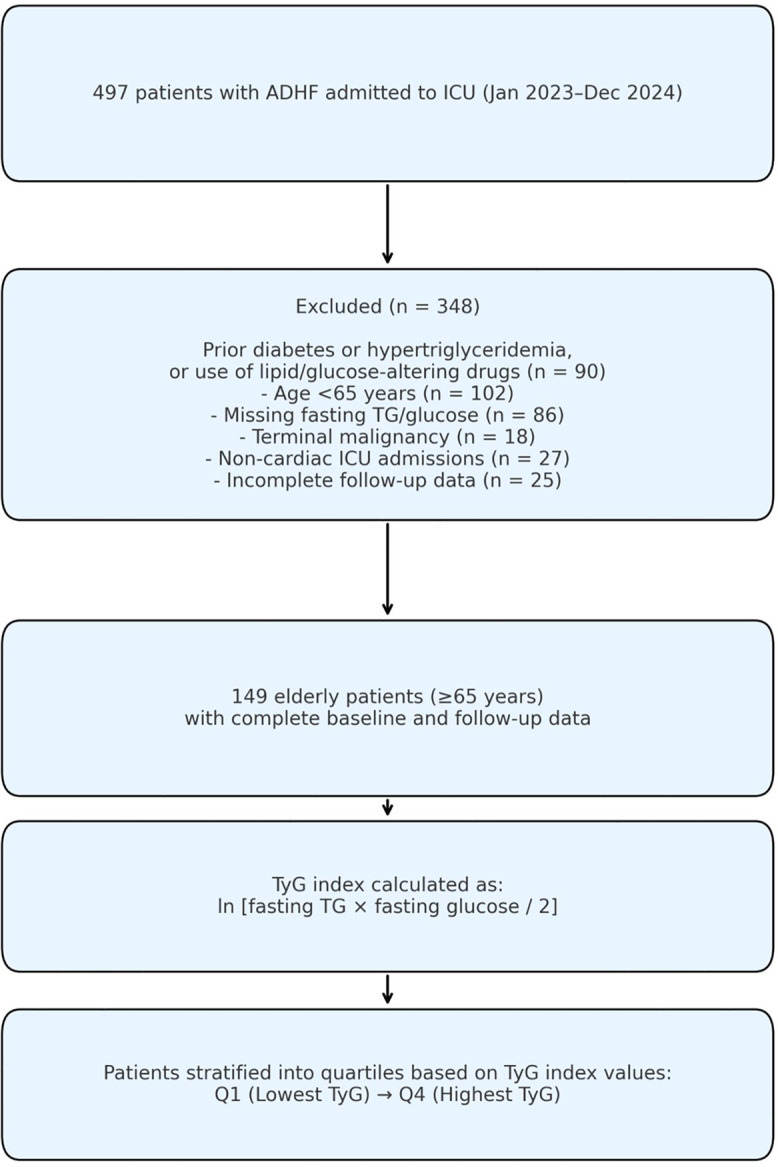
Flowchart of the study population enrollment. ADHF, Acute decompensated heart failure; ICU, Intensive care unit; TG, Triglyceride; TyG, Triglyceride-glucose index = ln [fasting triglyceride (mg/dL) × fasting glucose (mg/dL)/2

Between January 1, 2023 and December 31, 2024, a total of 497 consecutive patients admitted to the intensive care unit (ICU) with a diagnosis of ADHF were retrospectively screened. Patients were identified from the institutional electronic medical record system using ICD-10 codes corresponding to heart failure and acute decompensation. ADHF was defined according to the 2023 Focused Update of the 2021 ESC Guidelines for the diagnosis and treatment of acute and chronic heart failure (1). Accordingly, among these, 149 patients aged 65 years and older who met all inclusion criteria were enrolled in the final analysis (Figure 1).

To ensure data reliability and minimize bias, all clinical, biochemical, and echocardiographic information was cross-checked by two independent investigators. Laboratory measurements, including fasting triglyceride and fasting glucose levels, were obtained within the first 24 h of ICU admission. The triglyceride–glucose (TyG) index was calculated according to the following formula:

TyGindex=ln[fastingtriglyceride(mg/dL)×fastingglucose(mg/dL)/2]

This formula has been validated as a reliable surrogate marker of insulin resistance in cardiovascular and metabolic studies (4, 6).

Baseline demographic data (age, sex, body mass index), comorbidities (hypertension, coronary artery disease, chronic kidney disease, and atrial fibrillation), and medication history were recorded. Echocardiographic parameters—including left ventricular ejection fraction (LVEF), left atrial diameter, and diastolic function indices -were assessed according to current ASE echocardiography guidelines (10). Laboratory data included complete blood count, fasting glucose, renal and hepatic function tests, lipid profile, and natriuretic peptides. Additionally, clinical heart failure severity scores—namely Killip classification and New York Heart Association (NYHA) functional class—were recorded. Treatment strategies including heart failure medications, intravenous diuretics, inotropes, and vasodilator use were documented. Chronic kidney disease was defined according to KDIGO criteria as an estimated glomerular filtration rate <30 mL/min/1.73 m² (CKD stages 4–5), reflecting severe renal dysfunction, or a documented prior diagnosis of advanced CKD (11).

The duration of ICU stays and one-year follow-up outcomes were retrieved from institutional electronic databases. For patients with incomplete or missing follow-up information, supplementary data were obtained through detailed review of individual hospital records and discharge summaries. In cases where these sources were insufficient, structured telephone interviews were conducted with the patients or, when unavailable, with their first-degree relatives to confirm survival status and clinical outcomes.

#### Inclusion criteria

Patients were eligible for inclusion if they met all of the following criteria:

- Patients aged ≥65 years were included. Although geriatric medicine increasingly emphasizes ≥75 years, the ≥65-year cutoff remains standard in cardiovascular research and heart failure registries and was therefore adopted to ensure consistency with the existing literature.
- Hospitalization in the ICU with a confirmed diagnosis of ADHF
- Availability of fasting triglyceride and fasting glucose measurements obtained within the first 24 h of admission
- At least 12 months of clinical follow-up data
- Complete demographic, laboratory, and echocardiographic records available in the hospital database.

#### Exclusion criteria

Patients were excluded if they had any of the following:

- Known terminal-stage malignancy or life expectancy <3 months
- Admission to ICU for non-cardiac causes (e.g., trauma, sepsis, respiratory failure)
- Incomplete or missing data preventing TyG calculation
- Loss to follow-up or lack of one-year outcome data
- Patients with a prior diagnosis of diabetes or hypertriglyceridemia (very high fasting triglyceride levels ≥500 mg/dL), or those using lipid-lowering or glucose-altering medications that could confound baseline TyG levels, were excluded to minimize potential selection bias.

### Ethical considerations

The study protocol complied with the Declaration of Helsinki and received approval from the Karamanoğlu Mehmetbey University Faculty of Medicine Clinical Research Ethics Committee (date:10.09.2025, number: 23-2025/22). Owing to the retrospective design, the requirement for written informed consent was waived. All patient data were anonymized and handled confidentially.

### Statistical analysis

All statistical analyses were performed using IBM SPSS Statistics version 26.0 (IBM Corp., Armonk, NY, USA). Continuous variables were expressed as mean ± standard deviation (SD) for normally distributed data and as median (interquartile range, IQR) for non-normally distributed data, whereas categorical variables were presented as counts and percentages. Normality of distribution was assessed using the Shapiro–Wilk test. Comparisons between groups were performed using the independent-samples *t*-test or one-way analysis of variance (ANOVA) for normally distributed continuous variables and the Mann–Whitney *U* or Kruskal–Wallis tests for non-normally distributed variables, as appropriate. Categorical variables were compared using the Chi-square (*χ²*) or Fisher's exact test.

The TyG index was analyzed both as a continuous variable and after stratification into quartiles (Q1–Q4) based on its distribution within the study population. Quartile cut-off values were determined according to the empirical distribution of TyG values within the study sample (*n* = 149), representing the 25th, 50th, and 75th percentiles calculated directly from the observed dataset. The quartile cut-off values were as follows: Q1 (≤8.370), Q2 (8.371–8.770), Q3 (8.771–9.159), and Q4 (>9.160).

Receiver operating characteristic (ROC) curve analysis was performed to assess the predictive performance of the TyG index for one-year all-cause mortality, and the optimal cut-off value was determined using the Youden index. Univariate and multivariate Cox proportional hazards regression analyses were conducted to identify predictors of one-year mortality. All baseline demographic, clinical, laboratory, and echocardiographic variables presented in Table 1 were initially evaluated in univariate Cox regression analyses. Variables with a *p* value <0.05, as well as clinically established prognostic factors in heart failure, were entered into the multivariate Cox regression model. Accordingly, age, sex, left ventricular ejection fraction, hypertension, chronic obstructive pulmonary disease, cardiovascular disease, and chronic kidney disease were included *a priori* to ensure appropriate adjustment for confounding. Survival curves were generated using the Kaplan–Meier method and compared using the log-rank test.

**Table 1:** Comparison of clinical, laboratory, and demographic characteristics according to TyG quartiles.

Parameters	Q1	Q2	Q3	Q4	*p* value
Demographic Characteristics					
Age	73.50 [68.25–81.00]	81.00 [72.00–84.00]	79.00 [70.00–85.00]	81.00 [73.00–88.00]	0.065
Sex, Male *n* (%)	10 (26.3)	20 (54.1)	15 (40.5)	24 (64.9)	**0**.**006**
Laboratory findings					
Creatinine(mg/dL)	1.14 [0.97–1.52]	1.20 [0.95–1.74]	1.28 [1.01–1.45]	1.38 [1.14–1.82]	0.278
e-GFR (mL/min/1.73m²)	59.23 [38.31–68.73]	48.46 [33.62–67.00]	45.75 [38.73–70.76]	41.00 [26.00–54.79]	0.068
Sodium (mmol/L)	139.00 [136.25–141.00]	138.00 [136.00–140.00]	139.00 [136.00–140.00]	138.00 [134.00–139.00]	0.070
Potassium (mmol/L)	4.05 ± 0.53	4.29 ± 0.56	4.23 ± 0.55	4.57 ± 0.61	**0**.**002**
CRP (mg/L)	14.35 [7.75–23.42]	13.35 [4.15–53.68]	14.00 [6.72–66.95]	11.60 [4.70–56.90]	0.925
Glucose (mg/dL)	91.00 [84.00–105.50]	112.00 [97.00–124.00]	126.00 [105.00–152.00]	210.00 [142.00–242.00]	**<0**.**001**
LDL (mg/dL)	91.70 [67.25–104.15]	97.00 [76.60–119.60]	99.60 [80.80–116.20]	87.40 [74.40–111.10]	0.358
HDL (mg/dL)	37.00 [29.00–45.00]	41.00 [35.00–53.00]	41.00 [34.00–43.00]	40.00 [37.00–44.00]	0.132
Triglyceride (mg/dL)	70.50 [63.00–79.00]	94.00 [88.00–109.00]	121.00 [105.00–150.00]	192.00 [121.00–226.00]	**<0**.**001**
Total Cholesterol (mg/dL)	145.00 [114.00–158.75]	149.00 [133.00–189.00]	161.00 [147.00–187.00]	165.00 [143.00–196.00]	**0**.**003**
Hemoglobin (g/dL)	11.10 [10.10–12.93]	12.40 [10.50–14.10]	12.80 [12.20–14.40]	11.40 [10.50–13.40]	**0**.**014**
WBC (10³/µL)	7.17 [6.03–9.02]	7.05 [5.65–9.56]	9.56 [8.15–11.08]	8.32 [6.79–12.85]	**0**.**002**
Platelet (10³/µL)	206.50 [170.25–270.75]	226.00 [172.00–276.00]	238.00 [197.00–273.00]	255.00 [219.00–311.00]	**0**.**041**
Neutrophil (10³/µL)	5.62 [4.46–6.67]	5.66 [3.72–7.08]	6.94 [5.46–9.40]	6.12 [4.70–8.30]	**0**.**008**
Lymphocyte (10³/µL)	1.27 [1.02–1.60]	1.15 [0.94–1.84]	1.47 [0.77–1.95]	1.45 [1.17–2.09]	0.520
TyG Index	8.16 [8.02–8.26]	8.57 [8.49–8.67]	8.95 [8.86–9.08]	9.59 [9.40–9.96]	**<0**.**001**
Comorbidities, *n* (%)					
Hypertension	22 (57.9)	19 (51.4)	18 (48.6)	22 (59.5)	0.753
COPD	11 (28.9)	9 (24.)	8 (21.6)	8 (21.6)	0.865
Stroke	2 (5.3)	4 (10.8)	4 (10.8)	4 (10.8)	0.796
CKD	12 (31.6)	15 (40.5)	5 (13.5)	13 (35.1)	0.065
Cardiovascular findings					
Ejection Fraction (%)	40.00 [25.75–45.00]	40.00 [30.00–45.00]	35.00 [30.00–45.00]	40.00 [30.00–45.00]	0.732
Killip ≥3 (%)	10 (26.3)	8 (21.6)	12 (32.4)	16 (43.2)	0.191
NYHA ≥3 (%)	14 (36.8)	11 (29.7)	16 (43.2)	16 (43.2)	**0**.**050**
Hospitalizations and mortality					
Number of Hospitalizations (n)	3.00 [2.00–3.00]	2.00 [1.00–3.00]	2.00 [2.00–3.00]	2.00 [1.00–3.00]	0.103
Follow-up Duration (days)	390.50 [344.00–410.25]	395.00 [351.00–415.00]	371.00 [127.00–408.00]	337.00 [139.00–393.00]	**0**.**009**
Hospital Stay (days)	7.00 [6.00–8.00]	6.00 [6.00–8.00]	7.00 [6.00–8.00]	7.00 [5.00–8.00]	0.755
In-hospital mortality at first admission, *n* (%)	0 (0.0)	0 (0.0)	3 (8.1)	3 (8.1)	0.096
One Year Mortality, *n* (%)	11 (28.9)	10 (27.0)	15 (40.5)	20 (54.1)	0.061

Data are presented as median [interquartile range]. Continuous variables were compared using the Kruskal–Wallis *H* test, and normally distributed variables. Potassium levels were normally distributed and analyzed by one-way ANOVA. Categorical variables were presented as number (percentage) and compared using the Chi-square test. Bold *p*-values indicate statistical significance (*p* < 0.05). (CKD, Chronic Kidney Disease; COPD, Chronic Obstructive Pulmonary Disease; CRP, C-Reactive Protein; EF:Ejection Fraction; e-GFR, Estimated Glomerular Filtration Rate; HDL, High-Density Lipoprotein; LDL, Low-Density Lipoprotein; NYHA, New York Heart Association; TyG, Triglyceride–Glucose Index; WBC, White Blood Cell; *p*-value: statistical significance).

A two-tailed *p* value <0.05 was considered statistically significant.

## Results

### Participants and baseline characteristics

A total of 149 patients were included in the study. Patients were divided into four quartiles (Q1–Q4) based on the Triglyceride-Glucose (TyG) index, and their basic demographic, laboratory, and clinical characteristics were compared. Patients' age, renal function, electrolytes, inflammatory markers, hematological parameters, metabolic profile, and cardiac indicators were evaluated according to TyG quartiles.

Significant differences were found between TyG quartiles in glucose, triglycerides, total cholesterol, white blood cell (WBC), neutrophil, platelet, and hemoglobin levels (all *p* < 0.05). All of these parameters were higher in the highest TyG group (Q4). Follow-up duration showed a significant decrease as the TyG level increased (*p* = 0.009). No significant differences were observed between variables such as ejection fraction, sodium, creatinine, estimated glomerular filtration rate (eGFR), and length of hospital stay (*p* > 0.05).

When examining the distribution of categorical variables according to TyG quartiles, the proportion of female patients increased significantly as the TyG level increased (*p* = 0.006). Additionally, NYHA functional class showed a significant association with TyG, with an increased proportion of patients in the high TyG group having NYHA class ≥3 (*p* = 0.050). Although chronic kidney disease (CKD) and one-year mortality rates showed an increasing trend with TyG, they did not reach statistical significance (*p* = 0.065 and *p* = 0.061, respectively). No significant differences were observed between TyG groups in terms of hypertension, chronic obstructive pulmonary disease (COPD), cerebrovascular disease (CVD), Killip class, and in-hospital mortality (all *p* > 0.05) (Table 1).

When comparing the lowest (Q1) and highest (Q4) TyG quartiles, glucose, triglycerides, total cholesterol, WBC, neutrophil, and platelet levels were found to be significantly higher in the Q4 group, while the follow-up period was shorter (*p* < 0.05 for all). No statistically significant difference was found in hemoglobin levels (Table 2).

**Table 2:** Pairwise comparison (Mann–Whitney U) between lowest (Q1) and highest (Q4) TyG quartiles and kruskal–wallis *p*-values.

Variable	Q1 Mean Rank	Q4 Mean Rank	Direction (Q4 vs. Q1)	Kruskal–Wallis *p*
Glucose (mg/dL)	20.78	**55**.**69**	↑ Higher in Q4	**<0**.**001**
Triglycerides (mg/dL)	19.97	**56**.**51**	↑ Higher in Q4	**<0**.**001**
Total cholesterol (mg/dL)	29.62	**46**.**61**	↑ Higher in Q4	**0**.**003**
WBC (×10⁹/L)	32.68	**43**.**46**	↑ Higher in Q4	**0**.**002**
Neutrophil (×10⁹/L)	35.01	**41**.**07**	↑ Higher in Q4	**0**.**008**
Platelet (×10⁹/L)	31.66	**44**.**51**	↑ Higher in Q4	**0**.**041**
Hemoglobin (g/dL)	37.17	38.85	≈ No difference	**0**.**014**
Follow-up duration (days)	**45**.**18**	30.62	↓ Shorter in Q4	**0**.**009**

Mean ranks were obtained from the Mann–Whitney *U*-test (comparison between Q1 and Q4). Directional arrows (↑/↓) indicate the group with higher median values. All *p*-values are derived from the Kruskal–Wallis *H* test. Bold *p*-value: statistical significance (WBC, White Blood Cell).

### ROC analysis

**Figure 2:**
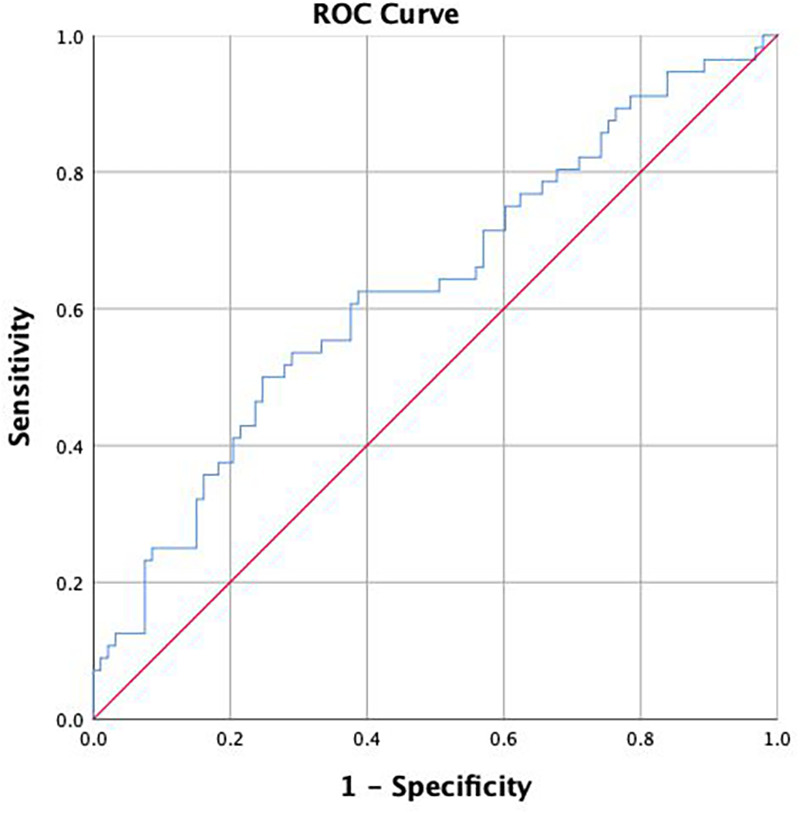
ROC analysis of TyG Index for prediction of mortality. Area under the curve (AUC) = 0.72; 95% CI: 0.61–0.83; *p* = 0.001. Optimal cut-off = 8.70 (Youden index).

The TyG index demonstrated moderate discriminative ability for one-year mortality (AUC = 0.72, 95% CI: 0.61–0.83, *p* = 0.001). An optimal cutoff value of 8.70 was identified, providing a sensitivity of 62% and specificity of 55%, with a Youden's J index of 0.17. These findings indicate that higher TyG levels are significantly associated with increased mortality risk and may serve as a clinically useful biomarker (Figure 2).

In the sensitivity analysis using the ROC-derived threshold of 8.70, the mortality rate was higher in the high TyG group compared with the low TyG group (44.9% vs. 29.6%), indicating an increased mortality risk above this cutoff. This threshold was derived in patients aged ≥65 years hospitalized with acute decompensated heart failure and should be interpreted as population-specific rather than universally applicable.

The chi-square test revealed that this difference was borderline statistically significant (*χ*² = 3.71, *p* = 0.054). Fisher's exact test confirmed that this difference was unidirectionally significant (*p* = 0.039). These results indicate that mortality risk tends to increase in patients with TyG ≥8.70 and that this threshold value can be used as a clinically discriminative cutoff.

### Survival analysis

**Figure 3:**
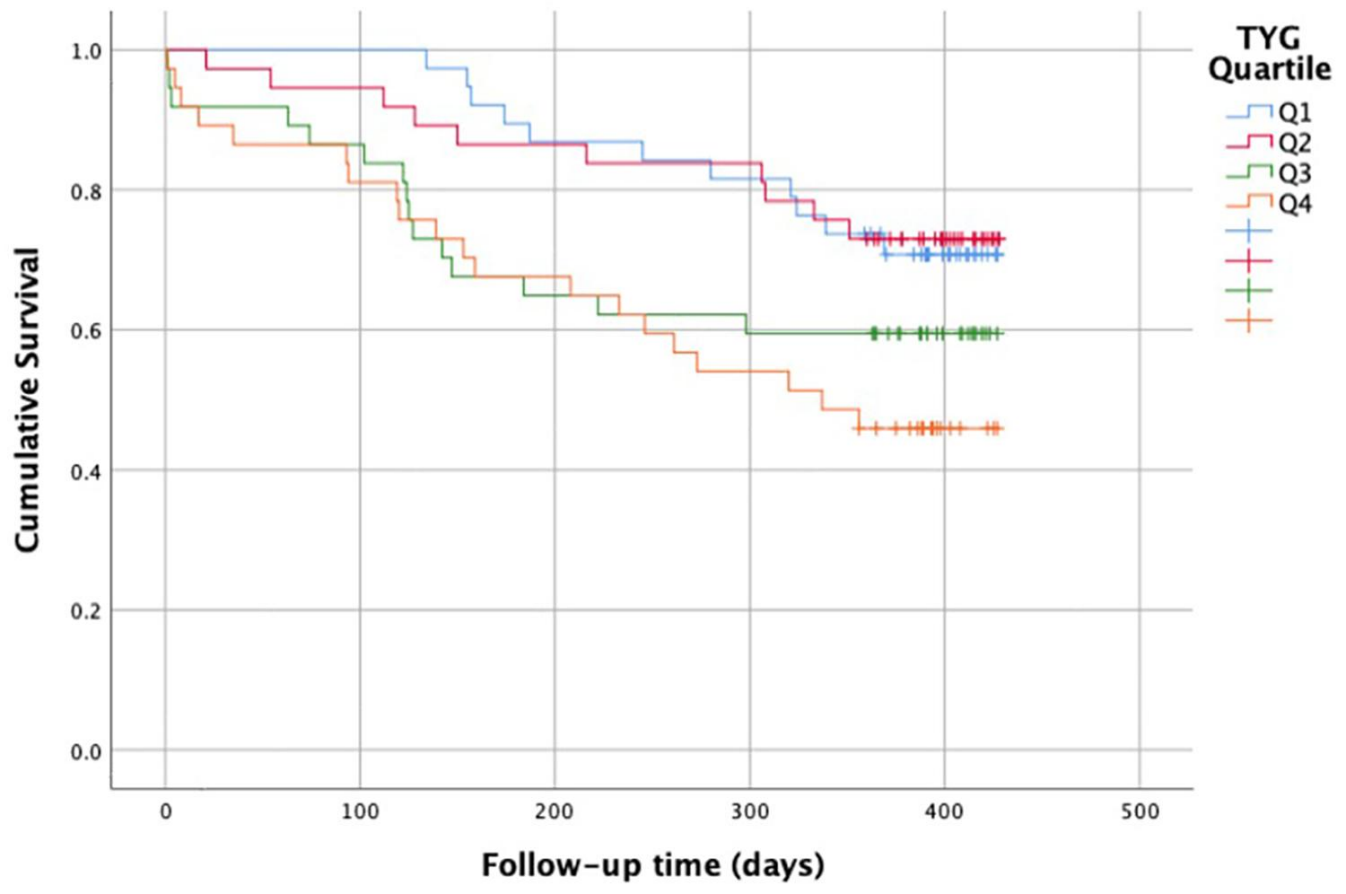
Kaplan–meier survival curves by TyG quartiles. Survival significantly decreased with increasing TyG levels (log-rank *p* < 0.001).

The Kaplan–Meier survival analysis demonstrated a significant decrease in survival time with an increase in the Triglyceride–Glucose (TyG) index (Log-rank *p* < 0.001). The median survival time in the lowest TyG quartile (Q1) was approximately 420 days, and this group had the highest survival rate (89.5%). In contrast, the median survival time in the highest TyG quartile (Q4) decreased to approximately 360 days, and the survival rate was found to be 45.9%. In the Kaplan–Meier curves, the curve for the Q4 group showed a marked decline in the early period, supporting the association between high TyG levels and increased mortality risk (Figure 3).

**Figure 4:**
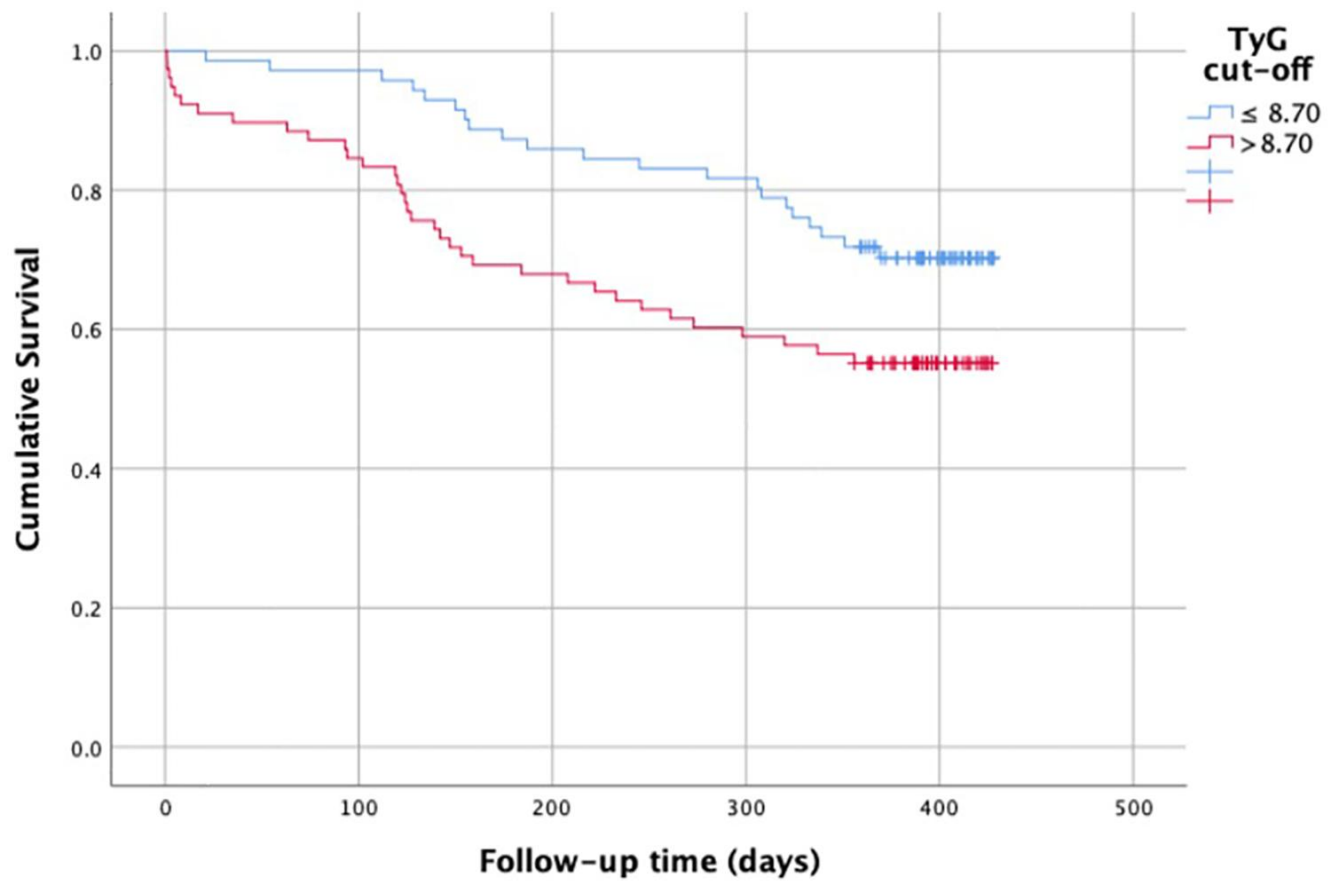
Kaplan–meier survival curves according to TyG Cut-off (8.70). Survival was significantly lower in patients with TyG >8.70 (log-rank *p* = 0.039).

According to the Kaplan–Meier curves created based on the TyG = 8.7 threshold obtained from the ROC analysis, the survival rate was significantly lower in patients with TyG >8.7; it was calculated as 70% vs. 55% at day 427 (log-rank *p* = 0.05) (Figure 4).

### Multivariable analysis

In the multivariate Cox proportional hazards analysis including age, sex, EF, hypertension, COPD, CVD, and CKD variables, TyG (continuous variable) was not found to be a significant independent predictor of mortality (HR 1.68; 95% CI: 0.70–4.00; *p* = 0.25). However, an approximately 68% increase in mortality risk was observed with increasing TyG levels. Similarly, TyG quartiles did not show significance (*p* = 0.868). No other variables were found to be significant independent predictors (all *p* > 0.05).

When TyG was entered into the multivariate Cox regression model as a categorical variable based on the ROC-derived cutoff (8.70), patients with TyG >8.70 had a significantly higher risk of one-year mortality (HR = 1.86, 95% CI: 1.03–3.37, *p* = 0.04).

### Subgroup analyses

In subgroup analysis, the effect of TyG on mortality was particularly pronounced in patients with low left ventricular ejection fraction (EF) (HR 1.91, 95% CI: 1.02–3.59, *p* = 0.04). The risk of all-cause mortality was significantly increased in cases with TyG >8.7 (HR 1.86, 95% CI: 1.03–3.37, *p* = 0.04). The interaction between age, sex, and TyG was not statistically significant (all *p* > 0.05) (Table 3).

**Table 3:** Subgroup Cox regression analysis for All-cause mortality.

Subgroup	HR (95% CI)	*p*-value	Interaction *p*
Age (65–74 vs. ≥75)	1.43 (0.77–2.66)	0.26	0.42
Gender (Male vs. Female)	1.58 (0.82–3.02)	0.17	0.38
EF (<40 vs. ≥40)	1.91 (1.02–3.59)	**0**.**04**	**0**.**03**
TyG Cut-off (>8.7 vs. ≤8.67)	1.86 (1.03–3.37)	**0**.**04**	—

EF, Ejection Fraction; TyG, Triglyceride–Glucose Index; bold *p*-value: statistical significance.

The predictive performance of the model was assessed using discrimination and reclassification metrics. Adding the TyG index to classic risk variables significantly improved the discriminative power of the model in predicting mortality. The area under the ROC curve (AUC) for the baseline model (age, gender, EF, HT, COPD, CVD, CKD) was 0.710 (95% CI: 0.641–0.776), while the AUC increased to 0.723 with the addition of the TyG variable. This increase in AUC was found to be statistically significant (*p* < 0.01). According to the reclassification analysis, the new model showed a net improvement of 27.3% in correct risk classification (NRI = 0.273, 95% CI: 0.213–0.334, *p* < 0.01) and a 1.1% increase in the average discrimination index (IDI = 0.011, 95% CI: 0.008–0.015, *p* < 0.01). These findings indicate that adding the TyG index to existing clinical variables provides additional prognostic value in predicting mortality risk (Table 4).

**Table 4:** Incremental predictive value of TyG for All-cause mortality.

Model	AUC (95% CI)	*Δ*AUC	NRI (95% CI)	IDI (95% CI)	*p*-value
**Model 1:** Age + Sex + EF + HT + COPD + CVD + CKD	0.710 (0.641–0.776)	—	—	—	—
**Model 2:** Model 1+ TyG	0.723 (0.658–0.789)	+0.013	0.273 (0.213–0.334)	0.011 (0.008–0.015)	**<0.01**

AUC, Area Under the Curve; CI, Confidence Interval; ΔAUC, Change in AUC; NRI, Net Reclassification Improvement; IDI, Integrated Discrimination Improvement; CVD, cerebrovascular disease CKD, Chronic kidney disease; TyG, Triglyceride–Glucose Index; EF, Ejection Fraction; HT, Hypertension; COPD, Chronic obstructive pulmonary disase; bold *p*-value, statistical significance.

## Discussion

This study comprehensively examined the prognostic effect of the Triglyceride-Glucose (TyG) index on all-cause mortality in geriatric patients hospitalized due to acute decompensated heart failure. Our findings show that high TyG values are significantly associated with both short-term and one-year mortality. These findings align with the current literature emphasizing the importance of the TyG index as a cardiometabolic risk marker.

The TyG index is an easily calculable surrogate marker of insulin resistance and is considered an important indicator of metabolic stress, particularly in the elderly patient population. Large cohort studies in recent years have reported that the TyG index is a strong predictor of cardiovascular events, atherosclerotic burden, and mortality (2, 3). A systematic review published by Gounden and colleagues in 2024 demonstrated that the TyG index is a valuable biomarker for assessing insulin resistance due to its simplicity in clinical practice and strong predictive performance (12).

Insulin resistance is increasingly recognized as a key mechanism linking metabolic dysregulation to frailty, cognitive decline, and physical impairment in older adults. Prediabetic states and insulin resistance have been associated with increased vulnerability and adverse outcomes in geriatric populations. Accordingly, the association between elevated TyG index values and increased mortality observed in our study may reflect not only metabolic risk but also a broader frailty-related phenotype in elderly patients hospitalized with acute decompensated heart failure, supporting the biological plausibility of our findings (13).

Cui et al. (14) reported that high TyG values in acute cardiac conditions may lead to cardiac dysfunction by increasing inflammatory activation and oxidative stress (14). In our study, it was observed that glucose, triglycerides, total cholesterol, WBC, neutrophil, and platelet counts increased significantly as the TyG level increased. These findings are consistent with previously defined pathophysiological mechanisms suggesting that TyG represents not only glycemic load but also inflammation and dyslipidemia. Furthermore, the findings obtained in our study are consistent with increasing evidence that the TyG index is associated not only with metabolic disorders but also with heart failure mortality. Furthermore, Zhao et al. (15) demonstrated that the TyG index is an independent predictor of subclinical atherosclerosis in the elderly population; this finding supports the observation in our study that patients with high TyG levels had a significantly higher inflammatory and metabolic profile (15). Again, these findings indicate that not only glycemic control but also triglyceride-based metabolic dysfunction is important in this patient group.

Adams-Huet et al. (16) demonstrated that TyG reflects not only glycemic load but also a unique lipotoxic profile, thereby significantly contributing to cardiometabolic risk classification (16). Additionally, Tao et al. (17) reported that high TyG levels are strongly associated with vascular stiffness and endothelial dysfunction; these pathophysiological processes provide biological links that explain increased mortality, particularly in the frail geriatric population (17).

Şaylık et al. (18) reported that the TyG index is significantly associated with mortality and complication risk in acute coronary syndromes (18). It is known that insulin resistance, inflammation, oxidative stress, and cardiometabolic dysfunction form a vicious cycle in older age. Our findings are consistent with this pathophysiological framework and suggest that elevated TyG during ADHF may be an indicator of acute metabolic burden in older individuals and a consistent prognostic tool across different cardiovascular subgroups.

The survival analyses in our study have more clearly demonstrated the effect of the TyG index on clinical outcomes. Kaplan–Meier curves show that survival time decreases significantly as the TyG level increases. It has been shown that survival is significantly lower in patients with high TyG levels. One-year survival was 89.5% in the Q1 group and 45.9% in the Q4 group. The fact that median survival was significantly reduced in the highest TyG quartile and that a rapid decline was observed in the early period supports the notion that TyG may be one of the determinants of short- and medium-term mortality risk.

In the ROC analysis, the AUC value was found to be 0.72, suggesting that TyG may be a moderately strong biomarker for distinguishing mortality. This result is consistent with the “moderate-to-high level of discriminatory power” of TyG in predicting vascular damage, atherosclerotic burden, mortality, and cardiometabolic risk reported in the literature by Tao and Gounden (12, 17). Furthermore, the significant increase in mortality rates in patients above the 8.70 cut-off value obtained from the ROC analysis (44.9% vs. 29.6%) suggests that TyG may offer a practically applicable threshold value in clinical settings. Accordingly, the moderate standalone ROC performance of the TyG index should be interpreted in light of its significant incremental prognostic value beyond conventional risk factors, as demonstrated by reclassification analyses. However, it should be noted that this cut-off value was obtained in a single center and limited sample size, and it needs to be validated in prospective studies for clinical use.

In our multivariate Cox regression analysis, the TyG index did not remain an independent predictor of one-year mortality (HR 1.68, *p* = 0.25). Several methodological and clinical factors may explain this attenuation of independent significance. First, the relatively modest sample size (*n* = 149) may have limited statistical power, particularly in multivariable models including several strong competing covariates. Second, dominant prognostic factors such as age, chronic kidney disease, reduced left ventricular ejection fraction, and overall comorbidity burden each included *a priori* in the multivariate model may have outweighed the incremental prognostic contribution of TyG, a phenomenon consistent with confounder dominance. To avoid multicollinearity, fasting glucose and triglyceride levels were not entered separately into the multivariate model, as they constitute the components of the TyG index. Therefore, the loss of independent significance is more likely attributable to overlapping prognostic information with established clinical variables rather than true biological irrelevance of the TyG index.

Moreover, mortality in geriatric ADHF is inherently multifactorial and is often heavily influenced by hemodynamic instability and multimorbidity, which may overshadow the prognostic impact of insulin resistance–related biomarkers. Notably, although TyG was not an independent predictor in the overall cohort, it demonstrated a significant association with mortality in patients with reduced ejection fraction (EF <40%) (HR 1.91, *p* = 0.04), suggesting that metabolic stress may exert a more pronounced clinical effect in this subgroup. This interaction may indicate that metabolic stress represented by TyG disproportionately affects patients with reduced EF, whose myocardium is already more vulnerable to energetic deficits, inflammation, and microvascular dysfunction. In such patients, increased metabolic load further impairs mitochondrial ATP generation and amplifies oxidative stress, accelerating myocardial deterioration. Supporting this interpretation, Zhao et al. (15) reported strong links between TyG and subclinical atherosclerosis, arterial stiffness, and endothelial dysfunction in elderly individuals—mechanisms that may partly explain the increased mortality observed in our high-TyG, low-EF patients (15). Together, these considerations indicate that the lack of statistical independence in the Cox model is more likely methodological than biological, a conclusion further reinforced by the clear gradient in survival curves and the significant NRI/IDI improvement after adding TyG to the baseline risk model.

One of the important contributions of this study is that the addition of the TyG index to clinical risk models significantly increases prognostic power. Indeed, in model performance analyses (increased AUC, NRI, and IDI), the addition of TyG to existing clinical parameters provides a significant improvement in risk prediction, strongly supporting the index's additional prognostic value. In particular, the NRI value showing a clinically meaningful reclassification contribution of 27% demonstrates that the TyG index provides information beyond classic risk factors. This result is fully consistent with the findings reported by Adams-Huet (16) that “TyG captures residual risk that traditional risk factors cannot identify by reflecting the combined metabolic effects of lipotoxicity and glycemic load” (16). Similarly, Gounden (12) demonstrated in their recent systematic review, which evaluated numerous prospective cohorts, that the TyG index provides a significant prognostic contribution to traditional parameters in predicting cardiometabolic risk (12). In our study, the achievement of meaningful reclassification gains with the addition of TyG to the model confirms that this index is a complementary biomarker that can strengthen clinical decision-making processes in geriatric ADHF populations. However, because no internal validation (e.g., bootstrapping or cross-validation) was performed, the improvements observed in ROC, NRI, and IDI analyses may carry a risk of overfitting.

Our study has several strengths. First, it is one of the first studies in the literature to focus exclusively on the geriatric ADHF population. The multifaceted analysis of TyG using robust statistical methods in a homogeneous geriatric ADHF population, the evaluation of its performance using reclassification analyses such as Kaplan–Meier, ROC, Cox, and NRI/IDI, and the provision of a clinically meaningful cut-off value enhance our study's contribution to the literature. Information about in-hospital and long-term mortality can be obtained using the TyG index, which is calculated using only two routine laboratory parameters, and this information can be used in patient discharge planning and determining long-term treatment strategies.

### Limitations

This study has several limitations that should be acknowledged. First, the study was conducted in a single center with a relatively small sample size (*n* = 149), which may limit the generalizability of the findings. Second, the retrospective design restricts the ability to establish a causal relationship between the TyG index and mortality. Third, the TyG index was calculated from single baseline measurements of fasting glucose and triglyceride levels during hospitalization; serial changes over time were not assessed. Fourth, we did not include other insulin resistance markers such as the HOMA-IR index or direct measures of insulin sensitivity for comparison. Fifth, long-term medication data (such as SGLT2 inhibitors, statins, or beta-blockers) and nutritional status were not comprehensively recorded, which might have influenced metabolic parameters.

The absence of systematically recorded long-term treatment data and standardized nutritional or frailty assessments represents an additional limitation, as these factors may modulate metabolic status and prognosis in elderly patients with acute decompensated heart failure.

Additionally, excluding patients with diabetes or lipid-lowering therapy may have systematically reduced TyG values and introduced a selection bias, potentially limiting the representativeness of the study cohort.

Although patients aged ≥65 years are commonly classified as elderly in cardiovascular and heart failure studies, contemporary geriatric definitions increasingly emphasize older age thresholds (≥75 years) and functional status rather than chronological age alone; this heterogeneity should be considered when interpreting our findings.

Despite these limitations, the study provides valuable preliminary evidence that the TyG index could serve as a simple, cost-effective marker for risk stratification in geriatric heart failure patients. Future multicenter studies with larger sample sizes and prospective designs are needed to validate these findings.

## Conclusion

In this study, elevated triglyceride-glucose (TyG) index levels were significantly associated with increased one-year mortality and reduced survival in elderly patients with acute decompensated heart failure. The TyG index showed moderate discriminative ability and provided incremental prognostic value beyond conventional risk factors, particularly among patients with reduced ejection fraction. Given its simplicity, cost-effectiveness, and availability from routine laboratory data, the TyG index may serve as a practical and reproducible biomarker for early risk stratification in geriatric heart failure management. Larger prospective multicenter studies are warranted to confirm these findings and to explore the clinical utility of TyG-guided risk assessment in daily practice.

## Data Availability

The original contributions presented in the study are included in the article/Supplementary Material, further inquiries can be directed to the corresponding author/s.
